# Clinical impact of multidrug-resistant bacteria in older hospitalized patients with community-acquired urinary tract infection

**DOI:** 10.1186/s12879-021-06939-2

**Published:** 2021-12-07

**Authors:** Manuel Madrazo, Ana Esparcia, Ian López-Cruz, Juan Alberola, Laura Piles, Alba Viana, José María Eiros, Arturo Artero

**Affiliations:** 1 Internal Medicine Department, Doctor Peset University Hospital, Avda. Gaspar Aguilar, n 90, 46017 Valencia, Spain; 2 Microbiology Department, Doctor Peset University Hospital, Avda. Gaspar Aguilar, n 90, 46017 Valencia, Spain; 3 Universitat de València, Avda. Blasco Ibañez, n 17, 46010 Valencia, Spain; 4 Department of Microbiology and Parasitology, Rio Hortega University Hospital, University of Valladolid, C/ Dulzaina, 2, 47012 Valladolid, Spain

**Keywords:** Older adults, Risk factor, Inadequate empirical antimicrobial therapy, Outcomes

## Abstract

**Introduction:**

Previous studies have described some risk factors for multidrug-resistant (MDR) bacteria in urinary tract infection (UTI). However, the clinical impact of MDR bacteria on older hospitalized patients with community-acquired UTI has not been broadly analyzed. We conducted a study in older adults with community-acquired UTI in order to identify risk factors for MDR bacteria and to know their clinical impact.

**Methods:**

Cohort prospective observational study of patients of 65 years or older, consecutively admitted to a university hospital, diagnosed with community-acquired UTI. We compared epidemiological and clinical variables and outcomes, from UTI due to MDR and non-MDR bacteria. Independent risk factors for MDR bacteria were analyzed using logistic regression.

**Results:**

348 patients were included, 41.4% of them with UTI due to MDR bacteria. Median age was 81 years. Hospital mortality was 8.6%, with no difference between the MDR and non-MDR bacteria groups. Median length of stay was 5 [4–8] days, with a longer stay in the MDR group (6 [4–8] vs. 5 [4–7] days, p = 0.029). Inadequate empirical antimicrobial therapy (IEAT) was 23.3%, with statistically significant differences between groups (33.3% vs. 16.2%, p < 0.001). Healthcare-associated UTI variables, in particular previous antimicrobial therapy and residence in a nursing home, were found to be independent risk factors for MDR bacteria.

**Conclusions:**

The clinical impact of MDR bacteria was moderate. MDR bacteria cases had higher IEAT and longer hospital stay, although mortality was not higher. Previous antimicrobial therapy and residence in a nursing home were independent risk factors for MDR bacteria.

## Introduction

Antimicrobial resistance has become a major worldwide healthcare problem and especially infections caused by multidrug-resistant (MDR) microorganisms as they have a huge clinical and economic burden [1, 2]. Although most of these infections are commonly associated with healthcare, MDR bacteria are also causing a growing number of community-acquired infections [3, 4].

Urinary tract infection (UTI) is among the most frequent causes of bacteremia and sepsis [5] and it is a very frequent cause of hospitalization due to infection in older adults [6]. Extended-spectrum beta-lactamases (ESBL) are enzymes produced by *Escherichia coli* and other bacteria that are common etiologies in UTI [7]. Simultaneous resistance to other antimicrobials is frequent since they are often encoded in the same plasmids that harbored the ESBL genes [8]. Therefore, MDR microorganisms are an increasing cause of UTI in both community-acquired and healthcare-associated infections [9–11] which could lead to a higher rate of treatment failure [12].

Previous studies have described some risk factors for MDR bacteria in UTI [13–15]. However, most of these studies are retrospective and include both nosocomial and community-acquired UTI. The clinical impact of MDR bacteria on older hospitalized patients with community-acquired UTI has not been broadly analyzed [16]. Therefore, we conducted a prospective study in older adults with community-acquired UTI admitted to hospital in order to identify risk factors for MDR bacteria and to know their clinical impact.

## Material and methods

Cohort prospective observational study of patients of 65 years or older consecutively admitted to an internal medicine ward at a university hospital diagnosed with community acquired UTI, from January 2016 to December 2019. Cases with a negative urine culture or a clinical syndrome compatible with any other condition after reviewing the case were excluded, as well as nosocomial or UTI cases transferred from the Intensive Care Unit (ICU). Epidemiological and clinical variables were collected by the authors following a protocol. All the cases were reviewed by two independent researchers (MM and AE) before being included in the study.

Multidrug-resistance (MDR) was defined according to an international expert proposal by Magiorakos et al. [17], as non-susceptibility to at least one agent in three or more antimicrobial categories (extended-spectrum penicillins, carbapenems, cephalosporins, aminoglycosides, and fluoroquinolones for gramnegative bacteria; and ampicillin, vancomycin, fluoroquinolones, fosfomycin and linezolid for grampositive bacteria). Extensively drug-resistance (XDR) was defined as non-susceptibility to at least one agent in all but two or fewer antimicrobial categories tested for a particular microorganism. SOFA and qSOFA scales were used according to their original definitions [18] and measured within 24 h of admission at the Emergency Department (ED). Community onset healthcare-associated UTI (HCA-UTI) was defined as a community onset UTI with any of the following criteria: (i) to have been admitted to an acute care hospital ≥ 48 h within 90 days previous to current hospital admission; (ii) to have received antimicrobial therapy within 90 days previous to admission; and (iii) residing in a nursing home [19]. IEAT was considered as the occurrence of infection that was not effectively treated at the time when the causative microorganism and its antimicrobial susceptibility were known. This included the absence of antimicrobial agents directed at a specific class of microorganisms and the administration of an antimicrobial agent to which the microorganism responsible for the infection was resistant [19].

Quantitative variables were compared using Student's t-test or analysis of variance (ANOVA) when the distribution was normal, or Mann–Whitney U-test when it was not normal. Qualitative variables were compared with chi square test and Fisher’s exact test. Multivariate analysis was performed using logistic regression, considering an α significance level of 0.05 for all tests. All tests were two sided. If any data was missing, a normal value was attributed for the calculation. The statistical package SPSS version 22 from IBM for Windows was used for the statistical analysis.

## Results

**Fig. 1:**
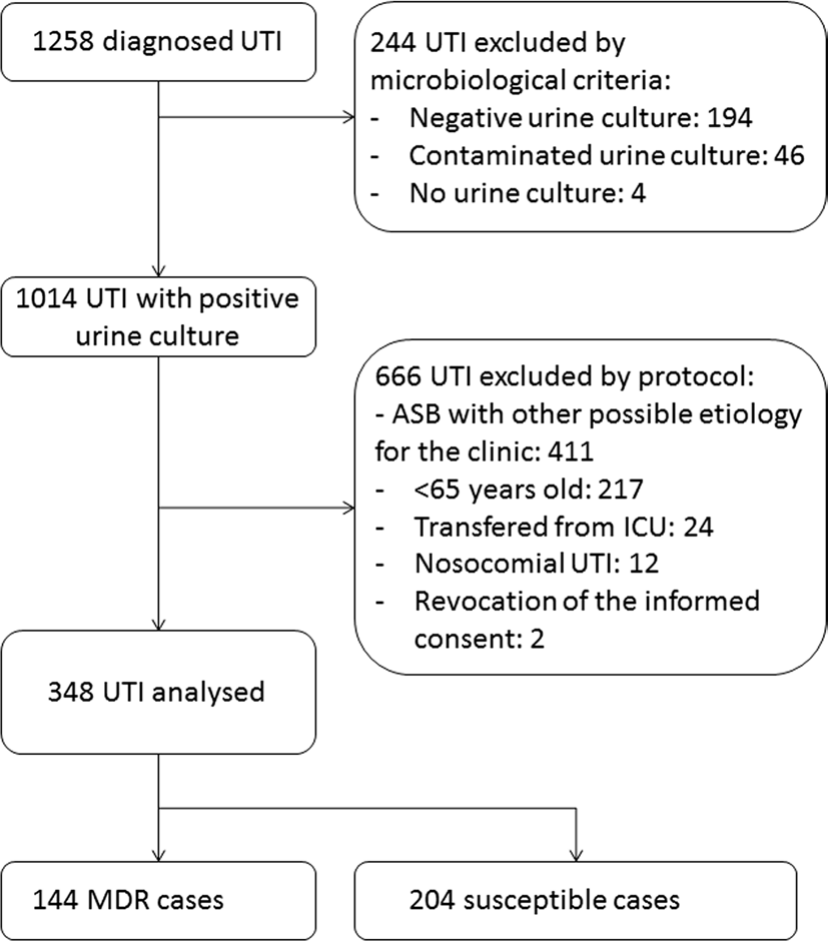
Flowchart of inclusion of hospitalized patients 65 years or older with community-acquired urinary tract infection

Out of 1258 patients diagnosed with community acquired UTI admitted to hospital during the period of study, 348 patients were included (Fig. 1). MDR bacteria caused 41.4% of the cases, including 7.8% cases with XDR bacteria. Mean age was 81 years, and 51.4% were male. Diabetes mellitus (37.4%), chronic kidney disease (32.5%) and dementia (29.9%) were the most frequent comorbidities. 52.9% of the patients were septic and 16% had septic shock on admission. Other epidemiological and clinical characteristics and outcomes may be seen in Table 1.

**Table 1:** Epidemiological and clinical characteristics and outcomes of community-acquired urinary tract infection in older patients according to multidrug-resistant or non-multidrug-resistant bacteria

	Total cases n 348	MDR bacteria n 144	Non-MDR bacteria n 204	p
Male sex, n (%)	179 (51.4)	82 (56.9)	97 (47.5)	0.102
Age (years), median [IQR]	81 [75–87]	80 [75–88]	81 [75–87]	0.719
Age ≥ 75 years, n (%)	274 (78.7)	111 (77.1)	163 (79.9)	0.595
McCabe ≥ 2, n (%)	268 (77)	113 (78.5)	155 (75.9)	0.607
Comorbidites				
Dementia, n (%)	104 (29.9)	52 (36.1)	52 (25.5)	0.043
Diabetes mellitus, n (%)	130 (37.4)	56 (38.9)	74 (36.3)	0.653
COPD, n (%)	51 (14.7)	20 (13.9)	31 (15.2)	0.761
CKD, n (%)	113 (32.5)	44 (30.6)	69 (33.8)	0.562
Cancer, n (%)	77 (22.1)	30 (20.8)	47 (23)	0.695
Indwelling urinary catheter, n (%)	81 (23.3)	40 (27.8)	41 (20.1)	0.122
HCA-UTI, n (%)	214 (61.5)	110 (76.4)	104 (50.9)	< 0.001
Previous hospitalization, n (%)	128 (36.8)	67 (46.5)	61 (29.9)	0.002
Previous antimicrobial therapy, n (%)	182 (52.3)	94 (65.3)	88 (43.1)	< 0.001
Nursing home residence, n (%)	28 (8)	20 (13.9)	8 (3.9)	0.001
Clinical characteristics				
APACHE II, median [IQR]	12 [9–17]	13 [9–17]	12 [9–17]	0.981
Fever, n (%)	239 (68.7)	98 (68.1)	141 (69.1)	0.907
RR ≥ 22, n (%)	71 (20.4)	23 (15.9)	48 (23.5)	0.105
Altered mental status, n (%)	158 (45.4)	76 (52.8)	82 (40.2)	0.021
SBP < 100, n (%)	62 (17.8)	30 (20.8)	32 (15.7)	0.255
qSOFA ≥ 2, n (%)	90 (25.9)	40 (27.8)	50 (24.5)	0.535
Sepsis (SOFA ≥ 2), n (%)	184 (52.9)	78 (54.1)	106 (51.9)	0.744
Septic shock-3, n (%)	56 (16)	25 (17.4)	31 (15.2)	0.657
Albumin, median [IQR]	3.1 [2.8–3.5]	3.0 [2.7–3.5]	3.2 [2.9–3.5]	0.063
Leukocytosis, median [IQR]	13,200 [9400–18575]	12,800 [9025–18425]	13,500 [9750–18600]	0.170
Polymicrobial UTI, n (%)	36 (10.3)	21 (14.6)	15 (7.4)	0.033
Bacteremia, positive/total blood cultures (%)	84/209 (40.2)	35/85 (41.2)	49/124 (39.5)	0.951
IEAT, n (%)	81 (23.3)	48 (33.3)	33 (16.2)	< 0.001
Outcomes				
In-hospital mortality, n (%)	30 (8.6)	11 (7.6)	19 (9.3)	0.699
30-day mortality, n (%)	44 (12.6)	20 (13.9)	24 (11.8)	0.624
Length of hospital stay (days), median [IQR]	5 [4–8]	6 [4–8]	5 [4–7]	0.029

MDR, multidrug-resistant; COPD, chronic obstructive pulmonary disease; CKD, chronic kidney disease; HCA-UTI, healthcare associated-UTI; RR, respiratory rate; SBP, systolic blood pressure; IEAT, inadequate empiric antimicrobial therapy

Hospital mortality was 8.6% and 30-day mortality was 12.6%, with no difference between the MDR and non-MDR groups. Mean length of stay was 5 [4–8] days, with a longer stay in the MDR bacteria group. IEAT was 23.3%, with statistically significant differences between groups (33.3% vs. 16.2%, p < 0.001).

MDR was associated with dementia, healthcare-associated UTI (including previous use of antimicrobials, previous admission at hospital and residing in a nursing home), and altered mental status at admission in the univariate analysis (Table 1). Healthcare-associated UTI variables, in particular previous antimicrobial therapy and residence in a nursing home, were found to be independent risk factors for MDR bacteria by multivariate analysis, but previous hospitalization was not (Table 2).

**Table 2:** Multivariate analysis by logistic regression of risk factors for multidrug-resistant bacteria producing urinary tract infection in older patients

	Univariate analysis p	OR (IC 95%)	Multivariate analysis p	OR (IC 95%)
Dementia	0.043	1.6 (1.1–1.7)	0.255	–
Altered mental status	0.021	1.4 (1.1–1.8)	0.197	–
Previous hospitalization	0.002	1.5 (1.2–1.9)	0.433	–
Previous antimicrobial therapy	< 0.001	1.7 (1.3–2.5)	0.003	1.7 (0.6–2.9)
Residence in a nursing home	0.001	1.8 (1.4–2.4)	0.005	2.7 (0.8–4.6)

*Escherichia coli* was the most frequent microorganism (56.4%), followed by *Klebsiella pneumoniae* (13.4%) and *Enterococcus faecalis* (8.5%), as shown in Table 3. The most frequent MDR bacteria were *E. coli*, *K. pneumoniae* and *Enterobacter cloacae* (60.5%, 12.6% and 6.7% of the total cases, respectively). There were no cases of panresistant bacteria. Multidrug-resistance in *E. faecalis* was low (6%), with just one case of resistance to Ampicillin and none to Vancomycin.

**Table 3:** Etiology of 348 cases of community-acquired urinary tract infection in older hospitalized patients according to multidrug-resistant and non-multidrug-resistant bacteria

	Total n 388	MDR bacteria n 147	Non-MDR bacteria n 241
Gramnegative bacteria, n (%)			
*Escherichia coli*	219 (56.4)	86 (39.26)	133 (60.73)
*Klebsiella pneumoniae*	52 (13.4)	20 (38.46)	32 (61.54)
*Pseudomonas aeruginosa*	28 (7.2)	7 (25)	21 (75)
*Klebsiella oxytoca*	11 (2.8)	3 (27.27)	8 (72.72)
*Enterobacter cloacae*	10 (2.6)	10 (100)	0
*Proteus mirabilis*	9 (2.3)	6 (66.67)	3 (33.33)
Other *Enterobacteriaceae*	12 (3.1)	8 (66.67)	4 (33.33)
Grampositive bacteria, n (%)			
*Enterococcus faecalis*	33 (8.5)	2 (6.06)	31 (93.94)
*Enterococcus faecium*	5 (1.3)	1 (20)	4 (80)
*Acinetobacter baumanii*	3 (0.8)	3 (100)	0
*Enterococcus gallinarum*	2 (0.5)	1 (50)	1 (50)
*Streptococcus agalactiae*	1 (0.3)	0	1 (100)
Fungi, n (%)			
*Candida* spp.	3 (0.8)	0	3 (100)

MDR, multidrug-resistant

Ampicillin showed the highest rate of resistance (75.6%), followed by ciprofloxacin (42.8%) and trimethoprim/sulfamethoxazole (40.5%). Resistance rates for Imipenem, Piperacillin/Tazobactam and Fosfomycin were low (4%, 6.4% and 15.6%, respectively), as well as resistance rate for Vancomycin among grampositive bacteria (4.3%).

## Discussion

Our findings in this study indicate that the clinical impact of MDR bacteria in older patients with community-acquired UTI was moderate. There was no difference in mortality and, despite IEAT being twice the percentage in the MDR bacteria group, the length of hospital stay was just 1 day longer.

In our study, MDR bacteria accounted for 41.4% of the cases of community-acquired UTI in hospitalized patients older than 65 years. This percentage is comparable to other results described in a similar setting (35.2% to 46.1%) [20–22], and higher than those of other studies on UTI outpatients (1.6% to 1.9%) [11, 23] or in the ED (6.7%) [24].

MDR cases were associated with IEAT (33.3% vs. 16.2%, p < 0.001) and a longer hospital stay (6 [4–8] vs. 5 [4–7] days, p = 0.029). Lee et al. [25], comparing MDR and non-MDR *Enterobacteriaceae* causing bacteraemic UTI, described similarly an effectiveness of empirical therapy of 43.5% vs. 93.9%, p < 0.001, and an increase in hospital stay (11.2 ± 6–62 vs. 9.27 ± 5.09 days, p < 0.001). In the same way, Tumbarello et al. [26] described an IEAT of 53.8% vs. 23.3% in a study with MDR and non-MDR *P. aeruginosa* UTI, as well as a longer hospital stay (48 vs. 22 days, p < 0.001).

In our study, in-hospital mortality was 8.6% and 30-day mortality was 12.6%, both higher than that found in other studies (2.2% to 3.3% and 6 to 9.4%, respectively) [20, 27–29], in which the mean age of the patients was lower. Nevertheless, our findings were lower than that found by Karve et al. (15% and 18.6%) [22]. MDR bacteria were not associated with mortality, neither in-hospital mortality nor 30-day mortality, similarly to that found in other studies with younger adults patients [25, 26]. Studies on the effects of antibiotic resistance on fitness often document fitness costs of varying severity [30], and this could explain the no difference in mortality between resistant and non-resistant infections in our study, although there was a difference in appropriate and inappropriate antibiotic treatment.

We would point out that in our study age and male sex were not associated with MDR bacteria, contrary to other studies on UTI, which included adult patients but not exclusively patients aged 65 or over [14, 16, 21]. It is a misconception that merely by being older, patients have more MDR infections. But this is not the case, as our findings suggest and in addition it can be seen in the works of Tumbarello et al. [26] and Faine et al. [24]. Comorbidities have been related to resistance in some studies [14, 25, 26], contrary to our results. In our study, only dementia and altered mental status on admission were significantly related to MDR in the univariate analysis, but not in the multivariate analysis, as in other studies with younger patients [16, 26].

Previous case history for healthcare related infection, in particular previous antimicrobial therapy or residence in a nursing home, was associated with MDR bacteria in our study. Previous antimicrobial therapy is one of the most important variables regarding multiresistance [14, 16, 21, 31] along with residing in a nursing home [14, 16, 21, 24, 31, 32]. Previous hospitalization has been related to resistance in some studies [14, 21, 31], but it was not significant in the multivariate analysis in our study, as well as in other works [16, 24, 32], The use of indwelling catheter has been traditionally related to the development of resistance [21, 26], but this was not the case in our results, as in other studies [16, 32].

ESBL-producing *Enterobacteriaceae* had a prevalence of 12.1% in our study, lower than the prevalence for ESBL-producing *Enterobacteriaceae* described in hospitalized patients in Europe (20%) [9] and North America (16.9%) [33], in studies with both community-acquired and nosocomial UTI. Our prevalence was similar to the 13.4% described by Smithson et al. [16] in older patients in the ED.

The resistance to ciprofloxacin was 42.8%, similar to other studies (46.8% to 47.7%) [34, 35], which warrants not using ciprofloxacin as empirical antimicrobial therapy [34, 36]. Fosfomycin is an antimicrobial drug used mainly in primary care, where low resistance rate is described [11]. However, in recent years, it has also been recommended for patients with MDR bacteria such as ESBL producing *Enterobacteriaceae* [4, 37], especially in older patients [7]. The low resistance rate in our case (15.6%) corroborates its usefulness as empirical treatment.

The main pathogens found in the urine culture, as expected, were *E. coli, K. pneumoniae* and *E. faecalis*. *E. coli* and *K. pneumoniae* were also the most frequent MDR bacteria (39.26% and 38.46% of the respective cases), similar to other studies describing gramnegative bacteria UTI [13] and *Enterobacteriaceae*-UTI [25]. There were only two cases of MDR *E. faecalis*, both in patients older than 85 years with healthcare associated infection, as described in other study [38], and none of them was resistant to vancomycin.

The main strength of our study is that it was conceived from a clinical point of view, embracing all the UTI in the population of our interest, and avoiding possible confounders had the study stemmed from the results of the urine cultures. It is not centered on a group of resistant microorganism such as *Enterobacteriaceae* [27], *Pseudomonas aeruginosa* [26] or gramnegative bacteria [9]; and it does not select only one type of the clinical spectrum of UTI, such as bacteremic UTI [1, 25]. We think that this patient orientated approach and overall view may help the clinician to treat better older patients with community-acquired UTI. Other strengths are the prospective design, as well as its rigorous selection of cases, conducted in every case by two independent researchers. The main limitation of this work is that it was carried out in a single center, with the bias that it represents, especially regarding etiology and resistance rates. Population in our study corresponds to older patients admitted to an internal medicine department. Indeed, it is a strength because this population is not always well represented in other studies [16], but it is also a limitation, as our findings could differ in other populations with UTI, such as patients attended in the ED or admitted to the ICU and those with nosocomial UTI. Another limitation is that we did not collect the antecedent of UTI caused by a MDR bacteria, which has been proved to be an important risk factor for antimicrobial resistance [34, 39, 40]. All in all, we believe that our findings add knowledge on risk factors for community-acquired UTI caused by MDR-bacteria and on their clinical impact in older adults, which can lead to better management of UTI in this population.

## Conclusion

In this prospective study in older patients with community-acquired UTI, the clinical impact of MDR bacteria was moderate. Cases caused by MDR bacteria had higher IEAT and longer hospital stay, although no higher mortality. MDR bacteria caused almost half of the cases of community-acquired UTI in our series, previous antimicrobial therapy and residence in a nursing home being independent risk factors.

## Data Availability

The datasets used and/or analysed during the current study are available from the corresponding author on reasonable request.
